# Developmental defects in ectodermal appendages caused by missense mutation in *edaradd* gene in the *nfr* mangrove killifish *kryptolebias marmoratus*

**DOI:** 10.1038/s41598-024-82276-z

**Published:** 2025-01-21

**Authors:** Hussein A. Saud, Paul A. O’Neill, Brian C. Ring, Tetsuhiro Kudoh

**Affiliations:** 1 Department of Pathological Analyses, College of Science, University of Basrah, Basrah, IQ Iraq; 2 College of Life and Environmental Sciences, University of Exeter, Biosciences, Exeter, EX4 4QD UK; 3 Department of Biology, College of Science & Math, Valdosta State University, 1500 N. Patterson St, Valdosta, GA 31698 USA

**Keywords:** *Edaradd*, *Eda*, *Kryptolebias Marmoratus*, *Aphanius dispar*, Developmental biology, Genetics, Diseases

## Abstract

The mangrove killifish, *Kryptolebias marmoratus*, can reproduce with self-fertilisation, offering a unique and useful genetic tool for generation of genetic mutants and quick identification of mutated genes. From an ENU-mutated mangrove killifish line R228, we have isolated a novel mutant line, no-fin-ray/*nfr* in which homozygous mutant of adult fish fin ray development is largely reduced. Illumina RNAseq with 3 embryos each from mutants, siblings and the parental WT strain Hon9 (only 9 embryos as total) identified a mutation in the *edaradd* in a highly conserved C-terminal death domain. Edaradd is known as a cytoplasmic accessory protein for the Ectodysplasin A (*EDA*) signalling pathway. To confirm the crucial role of *edaradd* during fin development, CRISPR RNAs were designed to suppress the gene in another killifish species, Arabian killifish. Indeed, Arabian killifish *edaradd* crispants showed a potent reduction of the fin development with 100% frequency. Furthermore, *EDA* crispants also showed identical phenotypes to that of *edaradd* crispants, confirming the fin defect in the mutants/crispants is caused by the signalling pathway of the *EDA* in the killifish species. These data demonstrate a powerful genetic approach using isogenic self-fertilising mangrove killifish as a tool for identifying mutants and their mutations, and revealed the crucial role of *edaradd* for the first time in the fish fin development.

**Supplementary Information:**

The online version contains supplementary material available at 10.1038/s41598-024-82276-z.

## Introduction

The self-fertilising mangrove killifish, *Kryptolebias marmoratus*, is a powerful model for a variety of genetics studies. There are two sexual forms, hermaphrodite and male. Hermaphrodites can generate fertilised eggs by internal self-fertilisation and they also can sexually reproduce by outcrossing male fish^1^. Laboratory strains were established by successive self-fertilisation for many generations (over 20 years) leading to the generation of highly isogenic and inbred lines^2,3^. Self-fertilisation and isogenicity makes this animal model extremely unique and useful for generating mutant lines and identifying the mutated gene(s)^4,5^. Firstly, when a mutated F1 fish contains a mutation in the genome, as this fish is a self-fertilising hermaphrodite possessing ovary and testis in the same body, the fish already produces eggs and sperms containing the same mutation. These fish are able to generate F2 homozygous mutant embryos within predictable Mendelian ratios^4^. Therefore, it is not necessary to separate groups of siblings in individual tanks and screen for carriers of the same mutation from two both sexes. Secondly, due to the isogenicity of the laboratory lines, genetic variations between two complementary chromosomes are highly limited, facilitating instant identification of mutated genes by a single run of sequencing with a small number of mutant, siblings and parental wild type strains, avoiding the need for genetic mapping^5^.

Ectodermal dysplasia (ED) is a hereditary disease causing defects in development of hair, teeth, secretory glands and digits^6^. From family genetic analyses, it has been discovered that one of the ED subtypes, hypohydrotic ectodermal dysplasia (HED) in patients that have a mutation in one of the three genes, ectodysplasin A/*eda*, *edar* or *edaradd*^7^. HED is diagnosed through chronic diseases including hypotrichosis, hypodontia and anhidrosis resulting in the reduction or absence of eccrine sweat glands, hair follicles and teeth, and defective formation of salivary, mammary and craniofacial glands^7^.

*Eda* encodes a TGF-beta super family signalling molecule. *EDA* protein binds to the receptor, *EDAR*, and in turn *EDAR* is associated with cytoplasmic protein *EDARADD*^8^. *Edaradd* encodes a protein that is considered as adaptor interacting with EDAR. EDAR/EDARADD complex transduce the signal via Table 2, TRAF6 and TAK1 to activate transcription factor NF-kappaB to target gene expression involved in ectodermal structural development^9^.

Similar to human patients, in the mouse, *edar*/*edaradd* mutations cause the disappearance of hair follicle and eccrine sweat gland and disorder in the glands of salivary and mammary craniofacial glands^10,11^. Furthermore HED mutant mice are affected by persistent rhinitis and otitis that arise from disorder in the mucociliary role^12^.

In fish species, *eda* and *edar* gene functions have been investigated in zebrafish and medaka. In zebrafish, *eda* and *edar* mutations (*nackt* and *finless* respectively) were identified by Harris et al. (2008) causing the disappearance of fins and scales and reduction in the number of pharyngeal teeth. Iida et al. (2014) also reported a nonsense mutation in *eda* in medaka fish called *alf *presenting coiled caudal peduncle without fin rays missing in all median and paired fins with a deformed skull, scales and teeth^13,14^.

Though the crucial role of *eda* and *edar* have been studied in these two model fish species, the role of *edaradd* was not fully known in the teleost fish. There is only one report of *edaradd *morpholino gene knock down study showing reduced development of the jaw and teeth^8^. However, the phenotype in the fin development was not reported in the loss of function of *edaradd* in fish species thus far. As morpholinos can be degraded after a few days of application it is conceivable that the phenotype in the fin development may not be fully affected by morpholino experiments. Therefore, loss of function studies involving genetic mutations of *edaradd* in fish models have been needed.

The first ENU mutagenesis applied to the self-fertilising mangrove killifish generated a variety of mutant phenotypes^3,4^. Among these, two lines R109 and R228 were sequenced and identified the mutations in the genes, *noto* and *msgn1*causing the phenotype of the shorttail/R109 and balltail/R228 respectively^5^. While maintaining the mutant lines, we identified another mutant phenotype that we named no-fin-ray (*nfr*) that was found from the R228 progenitor lineage. Here we report the *nfr* has a mutation in the *edaradd* gene and we show here a crucial role of the gene in the fins development.

## Materials and methods

### Maintenance of the mangrove killifish and arabian killifish

All mangrove killifish and Arabian killifish lines were reared and maintained in the Aquarit Resource Centre, University of Exeter. The mangrove killifish, R228 strain and parental wild type strain, Hon9 were maintained under the same laboratory conditions with 15ppt artificial sea water and aeration at 26° C ± 1, 12 h light: 12 h night photoperiod. Fish were fed on live *Artemia* once a day. Each fish was individually separated in 1.5 L containers. Water was changed once weekly. R228 strain was provided from Ring’s lab^4^. Eggs were collected by natural spawning from mature fish in both WT, mutant, and heterozygous carrier sibling strains. During the COVID lock down, the R228 line was discontinued and no longer available.

The Arabian killifish wild type strain was maintained with 35ppt artificial sea water in 40 L tanks as a small group with circulation and aeration at 26° C ± 1, 12 h light: 12 h night photoperiod. Egg chambers were placed at the bottom of the tank before the day of egg collection and eggs were collected by natural spawning in the morning 30 min to 1 h after the start of the light period.

## RNA extraction and analysis

Total RNAs were extracted used Qiagen RNeasy RNA purification kit from twelve embryos from the mutants, siblings and from wildtype (Hon9 strain) for the stages 16–18. Quality of RNAs were tested by Agilent RNA 6000 Nano Kit then sequenced using an Illumina HiSeq 2500 v3 next generation sequencer. Sequencing was conducted with 100 bp paired end reads on one lane. Sequencing adaptors firstly was trimmed then the low-quality flanks (< Q20) and short segments were removed using fastq-mcf v1.1.2–537. Assembly of transcriptomes were reconstructed by Trinity v2.2.0^13^. Variants were identified using KisSplice v2.4.0-p1^14^ into k-mer size of 53, then mapped to de novo transcriptomes by BLASTn v2.5.0^15^. Transcripts were annotated using blast-NCBI, and to select the best hits, threshold of 1e − 4 value to identify the suspected candidate genes.

## Skeletal staining with alcian blue and alizarin red

Samples were fixed with PFA, then washed with deionized water (2 times), and several times with DI water (2 h). Next water was replaced with Alcian blue (2 h) and placed into a series of ethanol washes (75%,50%,25%) each for ½ hr, and washed with 30% of tetraborate (2 times, 5 min. each.Next, the samples were transferred to Trypsin/sodium borate (0.12/30%) overnight, then washed with 2% KOH (2 times, 5 min. each) and replaced with Alizarin red (0.002% in 2% KOH) for one day. To bleach, embryos were placed into bleaching mixtures (3 of 0.5% KOH + 1 Glycerol + 10 µl H2O2) 3–4 h, and for clearance, the embryos were treated with a series of 0.5% KOH: Glycerol (1:1, 1:3, 100% Glycerol, 2 h each). Finally specimens were stored in 100% glycerol with a few thymol drops to prevent decay^16,17^.

### **Gene Knock out of*edaradd*and*eda*in the Arabian killifish**.

Two CRISPR RNAs were designed from *edaradd* (AAGAACTTTGCAAGCCGTTG and CTGCTAGAGCACCGGACCCA) and *eda* (ACCAACCACACGACCTTCCT and TTCAACACCTGCTACACGGC). Two crRNAs (final 50ng/ul each, Integrated DNA Technologies; IDT) and tracr RNA (final 100ng/ul, IDT), Cas9 nuclease protein (final 1ug/ul, IDT), 0.25% phenol red (Sigma) were dissolved in dilution buffer (IDT). Approximately 1 nl of the above crRNA mixture were injected into 1-cell stage Arabian killifish eggs. Embryos were cultured at 28’C for 11 days (the day of hatching) and were anaesthetised with tricaine and imaged using a Nikon SMZ1500 stereo microscope.

## Ethical declaration

All experiments were performed in accordance with approved protocols, relevant guidelines and regulations of the University of Exeter, Animal Welfare Ethical Review Board. All methods are reported in accordance with ARRIVE guidelines.

## Results

### **Compromised development in the fin**,** scale and mouth parts in*nfr*mutation**.

**Fig. 1:**
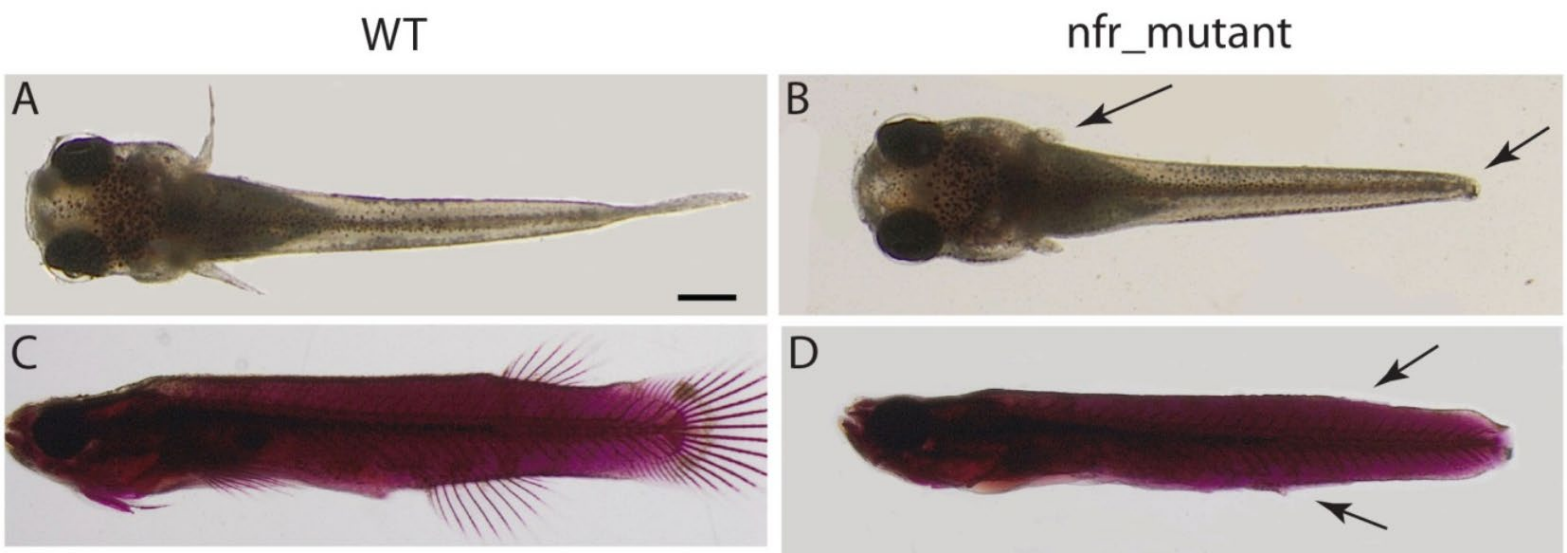
The mangrove killifish *nfr* mutant shows substantial reduction of the fin development in the post hatching larvae and juvenile fish. **A** and **C** are wildtype, **B** and **D** *nfr* mutants. **A** and **B** are post-hatched larvae and **C** and **D** are juvenile fish. Arrows refers to missing caudal and pectoral fins (**B**), dorsal and ventral fins (**D**). Both **C** and **D** are stained with alizarin red and alcian blue. Scale bar = 200 μm.

*Nfr* mutant presented clear phenotype showing deletion in all fins (Fig. 1). At early stages of development (specifically at stages 28 to 29) fins formation starts to emerge^18^.

**Fig. 2:**
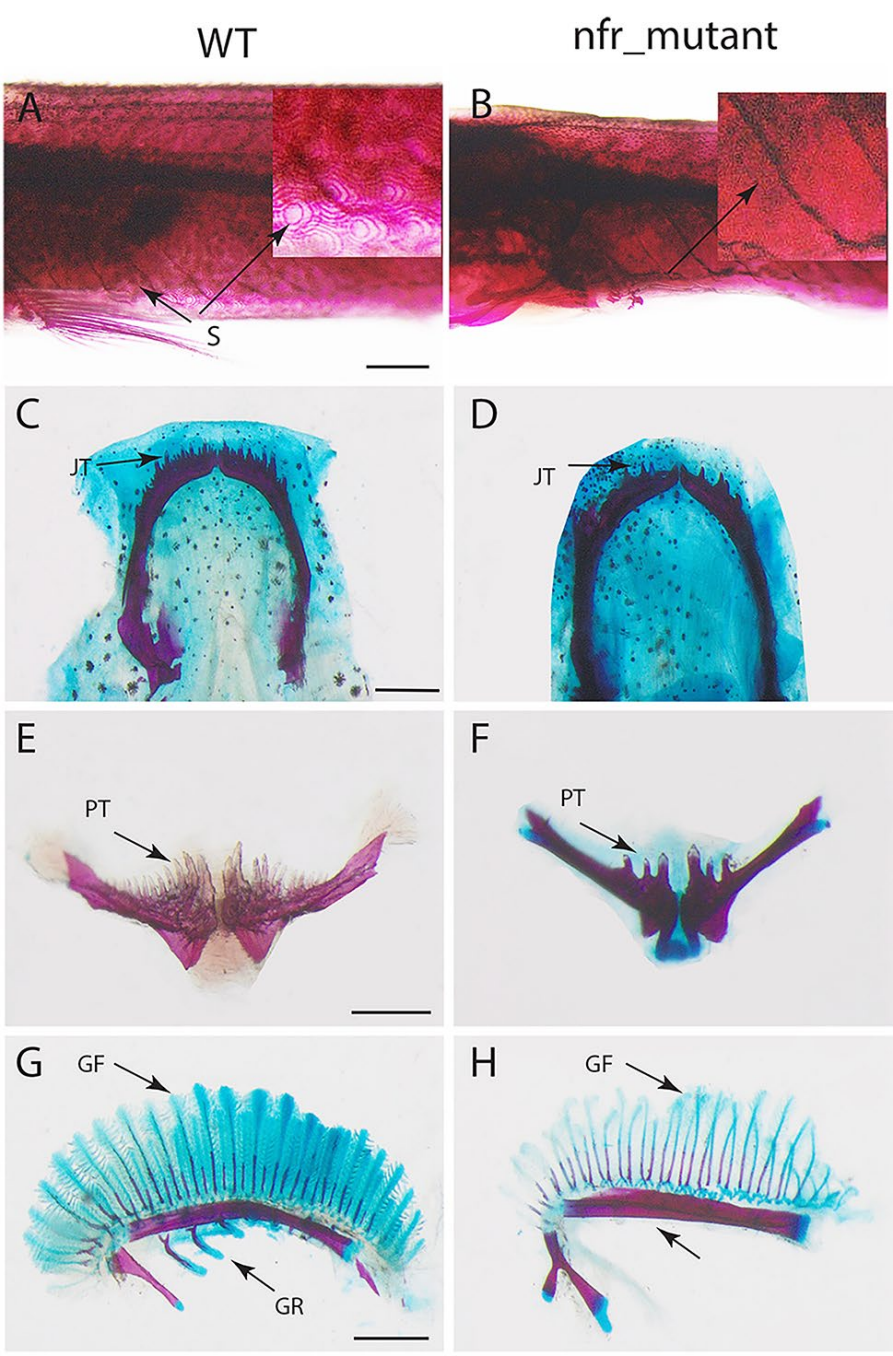
Scales, teeth and gills in mangrove killifish. **A**, **C**,**E**, **G** wildtype; **B**, **D** , **F**, **H** *nfr* mutant. S: scales (**A**), no scales in (**B**); **JT**: jaws teeth, in **D** and **F** number of teeth were reduced compared with wildtype (**C**, **E**); PT: pharyngeal teeth are reduced in H compared with wildtype (**G**); number of gill rackers (**GR**) and gill filaments (**GF**) are dramatically decreased. All parts stained with alizarin red and alcian blue. Scale bar = 200 μm.

The phenotype examination in the skin and mouth parts revealed scales were not visible with alizarin red staining and decreasing in the number of teeth in jaws and pharyngeal teeth (Fig. 2). It was observed remain the big teeth with missing the small one in both jaws and pharyngeal teeth. In addition, there was an obvious and dramatic decrease in the number of gill filaments and gill rakers.

### ***Nfr*is missense mutation of*edaradd***.

**Fig. 3:**
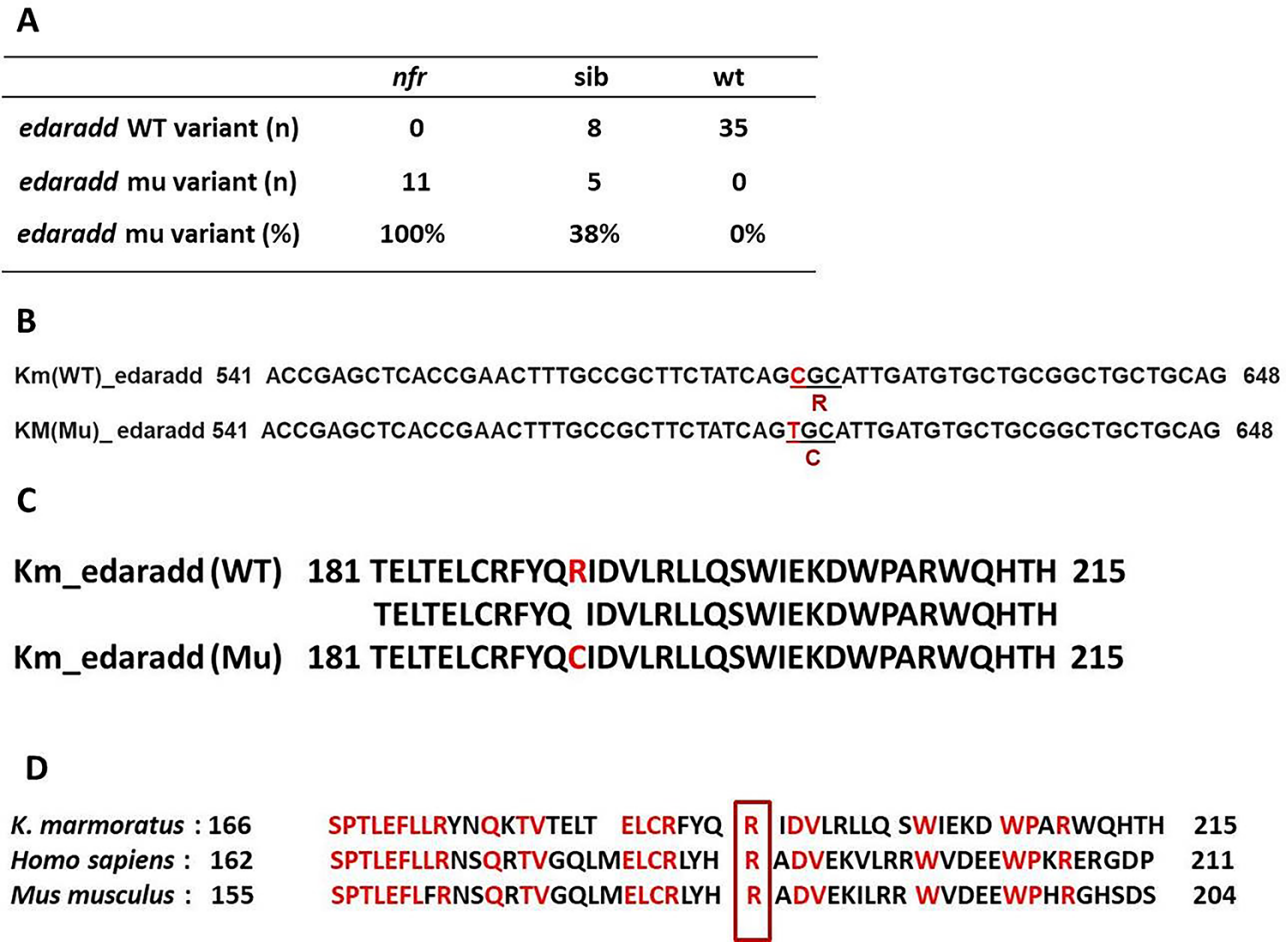
**A**. The mutation in the *edaradd* was 100% enriched in the *nfr* mutant embryos, 38% in the siblings and 0% in the WT embryos. **B**. Point mutation at loci 574 in nucleic acid base cytosine changed it to thymine in Km- *nfr* mutant. **C**. The resulting missense mutation changes the amino acid Arginine to Cysteine in the mutant. **D**. The mutation occurs in the highly conserved Arginine that is in the C-terminal death domain common to both the mangrove killifish (Km), thehuman and mouse.

To identify the *nfr* mutation, *nfr*, their sibling and WT embryos were analysed with RNAseq. three embryos from each category (WT, mutants and siblings) were sequenced with one lane of Illumina HiSeq 2500 100 bp paired end read. From the variations identified, the one which is 100% enriched in the mutant group, 0% in the WT group and intermediate number in the sibling group were searched, leading to the identification of 19 potential sequence variations. Among these, only seven variants were in a protein coding region: Three were synonymous variation and other four were non-synonymous. These four non-synonymous mutations were seen in the genes, *edaradd*,* herpud1*,* pacs1* and *nomo* (Supp Fig. 1). In the sibling group, if the variation is responsible for the phenotype, the WT sequence is expected to be twice abundant as the mutant. *Herpud1* and *pacs1* did not show such ratio implied that this was not responsible for the *nfr* mutant phenotype. The amino acid change of *nomo* was Ser to Thr, that can frequently occur in natural variation without causing deleterious phenotype. Consequently, *edaradd* remained as the best candidate for the *nfr* mutation (Fig. 3). In the *nfr* mutant, Arg 192 is replaced with Cysteine. Arginine 192 is within the death domain and is a highly conserved amino acid among orthologs in vertebrate species (Fig. 3). A missense mutation in a highly conserved amino acid in the functional domain suggested that the mutation would cause a severe loss of function of the protein.

### ***Edaradd*crispants in Arabian killifish phenocopies*nfr***.

**Fig. 4:**
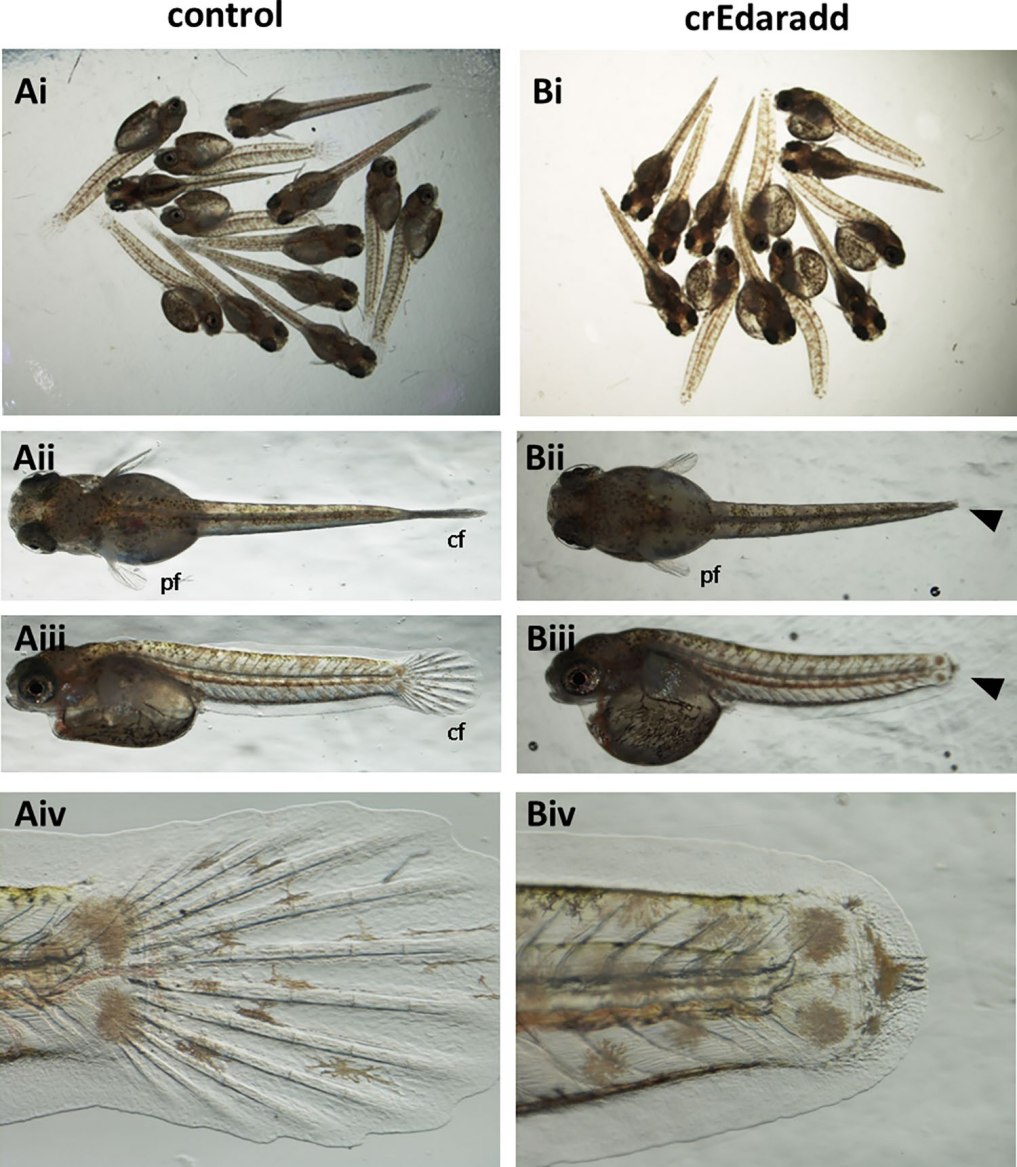
*Edaradd* crispants show loss of the caudal fin with 100% frequency. Mixture of two CRISPR RNAs for *edaradd* was injected into the 1-cell stage Arabian killifish eggs. Phenotypes were analysed at 11dpf (hatching stage) showing loss of the caudal fin (**cf**.) in all crispant embryos (**Bi** to **Bv**) as compared to WT controls (**Ai** to **Av**). At this stage, pectoral fin (**pf**) development was not significantly affected.

To confirm that suppression of *edaradd* can cause the no fin ray phenotype, two CRISPR RNAs were designed from the *edaradd* gene and administered to the Arabian killifish model. Due to internal self-fertilization prevalent in the mangrove killifish it is difficult to generate sufficient number of 1-cell stage embryos for CRISPR injections from a limited number of adult fish. So, we used the Arabian killifish to recapitulate the *nfr* phenotype. Indeed, the crispants of *edaradd* in the Arabian killifish showed a clear loss of caudal fin 11dpf embryos/larvae (the hatching stage) with 100% frequency (Fig. 4). At this stage, defects in the pectoral fins were not that obvious.

### ***Edaradd*loss of function shows similarity to loss of*eda***.

**Fig. 5:**
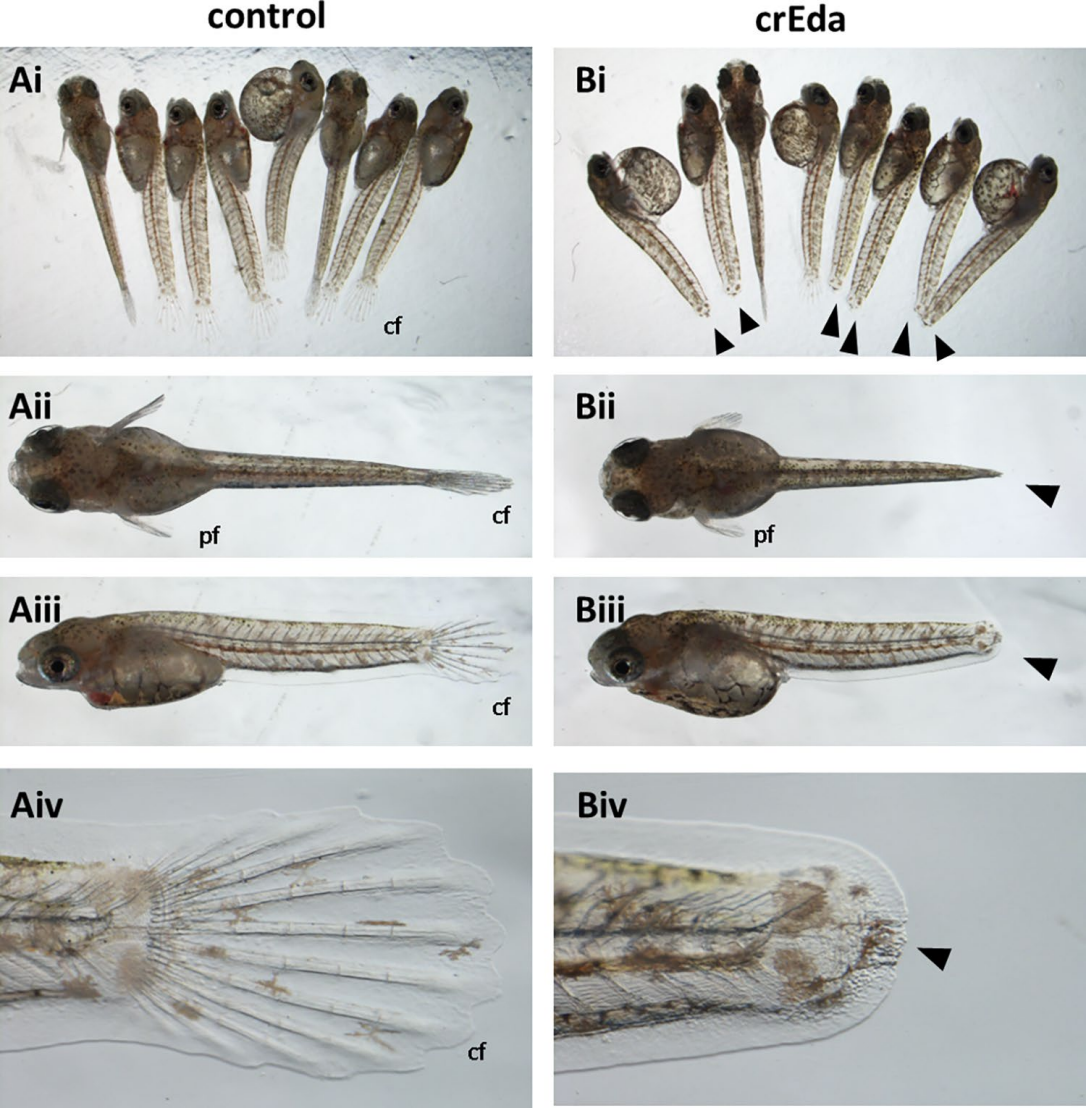
*Eda* crispants show loss of the caudal fin with 70–80% frequency (**Bi** to **Biv**) versus WT controls (**Ai** to **Aiv**). A mixture of two CRISPR RNAs for *edar* was injected into the 1-cell stage Arabian killifish eggs. Phenotype were analysed at 11dpf (hatching stage) showing loss of the caudal fin (**cf**.) in the majority of crispant treated embryos. At this stage, pectoral fin (**pf**) development was not significantly affected.

It has been known that *edaradd* act as the downstream cytoplasmic adaptor molecule of the *EDA*/*EDAR* signalling pathway. To confirm that *edaradd* is indeed acting along this pathway in the teleost species during fin development, the upstream gene, *eda*, was also suppressed by injecting CRISPR RNAs in the Arabian killfish. Although the percentage of the embryos showing abnormalities were lower in the *eda* crispants (70 to 80%, Fig. 5) as compared to the *edaradd* crispant treatment group (100%), we observed an indistinguishable morphological change of caudal fin loss at the hatching stage in both treatments (Figs. 4 and 5). Not only the loss of the caudal fin, but also the morphological details such as abnormal accumulation of pigment cells at the growing edge of the caudal fin was equally observed in the crispants of the two genes. As seen in the *edaradd* and *eda* crispants also did not show a significant reduction of the pectoral fin at the hatching stage.

## Discussion

Here we report identification of the *no fin ray* mutant from a previous ENU mutagenesis screen utilizing the self-fertilizing mangrove killifish. The homozygous *nfr* fish are viable and can spawn eggs as normal with self-fertilisation with 100% frequency of *nfr* phenotype in the offspring forming a true breeding clonal mutant line. RNAseq analysis of the *nfr*, sibling and Hon9 (parental WT strain) with a small number of mixed embryos (3 duplicates samples) was sufficient to narrow down all variations to a single non-synonymous mutation that occurred in the *edaradd* encoding Arginine 192. The resulting missense mutation changed Arginine to Cysteine in the C-terminal death domain. Using the same method employed previously in the characterization and identification of two other mutants, *shorttail* and *balltail* as the mutations of *noto* and *msgn1*respectively^5^, this method is very powerful for rapid identification of mutant alleles. This is strong evidence supporting the use of isogenic mangrove killifish strains as extremely useful for identifying mutations as the number of polymorphic variations are highly reduced. Even in the protein non-coding regions, we only identified 16 variants, suggesting that more subtle mutation phenotypes that are caused by mutations in the non-coding regions or promoter/enhancer regions may also be identified and characterised in future forward genetic screens.

This is the first report showing that an *edaradd* mutation causes the loss of fin phenotype in teleost fish species and we have confirmed it in two species, the mangrove killifish and Arabian killifish. The phenotype that was observed in this work with *edaradd* mutations was highly similar to the one reported in zebrafish with *eda* and *edar*^19^ and medaka with *eda*^20^. These findings indicate the pathway is widely conserved in teleost fish species. And in a broader context, the pathway is also conserved between fish and mammals^21^.

To determine if the *nfr* allele functions similarly in another species, a crisper knock out of *edaradd* was performed in another genetic model species (Arabian killifish) belonging to the Cyprinodontidae family of Teleost fish. These experiments demonstrated that a similar phenocopy of *nfr* mutant could be recapitulated in a reverse genetics approach. The Arabian killifish *edaradd* crispant revealed disappearance of caudal, dorsal and ventral fins whereas the pectoral still developed at the hatching stage, albeit with an abnormal shape.

Of further interest is the observation that caudal fin development was suppressed with loss of the fin rays despite the size of pectoral fin was not significantly affected at this stage (hatching stage). It should be noted that in the caudal fin, although fin rays did not develop, the epidermal layer of the caudal fin fold seems fully developed (Fig. 4). This may suggest that the role of the *eda*/*edar*/*edaradd*pathway is not directly involved in the fin epidermal development but upstream. Considering this evidence, although all cell lineages are missing in the caudal fin ray, it is possible that the role of this pathway is highly specific to a cell lineage such as cartilage cell differentiation as previously suggested and defects in other cell lineage may be a secondary consequence of the defect^8,12,20^. Considering that early stages of fin development such as fin bud formation are not affected and epidermal fin fold development is not affected either, this may explain why the size of the pectoral fin at the hatching stage is not affected by the *edaradd* crispants.

Here we report a homozygous viable mutant strain in the mangrove killifish and identification of the mutated gene. In many cases, it is conceivable that homozygous viable mutants are not easy to maintain because even though they are viable, active mating behaviour might be compromised. However, by using a self-fertilising animal, it is possible to avoid the step of mating, and lines can be established with relative ease. We have demonstrated this in subsequent genetic screens and more recently other researchers have added to repertoire of genetic tools in the mangrove killifish such as high fecundity genetic line, *kissylip*^23^. It is also possible to analyse subtle phenotypes (e.g. learning difficulty, aggression, altered facial structure, microcephaly) using this isogenic self-fertilising model. Genetic screening of such weak phenotypes at adult stages is easier as individual variation can be screened among established clonal lines. Therefore. this model is poised for future studies of natural, mutagen induced, and CRISPR mediated genomic variants.

## Electronic supplementary material

Below is the link to the electronic supplementary material.

Supplementary Material 1

## Data Availability

Sequence data that support the findings of this study have been deposited in the NCBI GEO (GEO Submission GSE273026).
