# Supplementary material for: Developmental defects in ectodermal appendages caused by missense mutation in edaradd gene in the nfr mangrove killifish kryptolebias marmoratus
Source: Sci Rep. 2025 Jan 21;15:2660. doi: 10.1038/s41598-024-82276-z (PMC11751105; doi:10.1038/s41598-024-82276-z)
Supplement: Supplementary file 1 — Supplementary Material 1 [file 41598_2024_82276_MOESM1_ESM.pdf]

		wt variant			mut variant				
Bubble_ID	total_reads	wt	mut	sib	wt	mut	sib	AA change	Gene name
16962_0	319	62	0	4	0	89	76	Asp > Asn	*herpud2*
24487_0	104	24	0	11	0	29	8	Val > Ala	*pacs1*
26940_0	668	171	0	105	0	188	28	Ser > Thr	*nomo*
55370_0	57	19	0	8	0	11	3	Arg > Cys	*edaradd*

Supplementary Figure 1
Three embryos each from WT, mutant and sibling were sequenced by RNAseq. Among all read, four genes were identified having no- synonymous mutations with 100% in wt, 0% in mutant (mut) and a number in between in siblings (sib).
